# Detection of Congestive Heart Failure and Myocardial Dysfunction in Cats With Cardiomyopathy by Using Two-Dimensional Speckle-Tracking Echocardiography

**DOI:** 10.3389/fvets.2021.771244

**Published:** 2021-11-15

**Authors:** Ryohei Suzuki, Takahiro Saito, Yunosuke Yuchi, Haruka Kanno, Takahiro Teshima, Hirotaka Matsumoto, Hidekazu Koyama

**Affiliations:** Laboratory of Veterinary Internal Medicine, School of Veterinary Medicine, Faculty of Veterinary Science, Nippon Veterinary and Life Science University, Musashino, Japan

**Keywords:** cat, feline, heart, hypertrophic cardiomyopathy, myocardial function, restrictive cardiomyopathy, right heart function, strain

## Abstract

Congestive heart failure (CHF) is a life-threatening condition in cats with cardiomyopathy. We hypothesized that myocardial dysfunction may induce progression to CHF pathophysiology in cats with cardiomyopathy. However, no previous studies have evaluated the involvement of myocardial dysfunction in cats with CHF. In this study, we aimed to evaluate the relationship between CHF and myocardial function assessed using two-dimensional speckle-tracking echocardiography (2D-STE). Sixteen client-owned healthy cats and 32 cats with cardiomyopathy were enrolled in this study. Cats were classified into three groups: healthy cats, cardiomyopathy without CHF (CM group), and cardiomyopathy with CHF (CHF group). Left ventricular (LV) longitudinal and circumferential strains (SL and SC, respectively), and right ventricular (RV) SL were measured using 2D-STE. Logistic regression analysis was performed to assess the relationship between CHF and echocardiographic variables, including 2D-STE. Results comparing the healthy cats and CM vs. CHF groups showed that increased left atrial to aortic diameter ratio and decreased LV apical SC were significantly associated with the existence of CHF (odds ratio [95% confidence interval]: 1.40 [1.16–1.78] and 1.59 [1.06–2.36], respectively). Results comparing the CM vs. CHF group showed that increased end-diastolic RV internal dimension and decreased RV SL were significantly associated with the existence of CHF (odds ratio: 1.07 [1.00–1.13] and 1.34 [1.07–1.68], respectively). Left atrial enlargement and depressed LV apical myocardial function may be useful tools for predicting the progression to CHF in cats. Furthermore, RV enlargement and dysfunction may lead to the onset of CHF in asymptomatic cats with cardiomyopathy.

## Introduction

Congestive heart failure (CHF) is a life-threatening condition in cats with heart disease (1). In veterinary medicine, hypertrophic cardiomyopathy (HCM) and restrictive cardiomyopathy (RCM) are common heart diseases that progress to CHF in cats (2). Both diseases are thought to be primarily caused by myocardial lesions, and may involve deterioration of myocardial function. We hypothesized that myocardial dysfunction may lead to increased left ventricular (LV) filling pressure and thereby induce progression to CHF pathophysiology. However, no previous studies have evaluated the involvement of myocardial dysfunction in cats with CHF.

Currently, some echocardiographic parameters have been used to evaluate disease progression in cats with cardiomyopathy, as well as to detect CHF pathophysiology (1, 3–5). However, most conventional echocardiographic parameters may be affected by loading conditions, heart rate, and technical limitations (such as Doppler angle limitation), which may inhibit the early detection of CHF (5). Recently, two-dimensional speckle-tracking echocardiography (2D-STE) has enabled the quantitative assessment of intrinsic myocardial function, and has been reported for myocardial assessment in feline patients (6–12). However, no previous studies have evaluated the relationship between variables of myocardial function using the 2D-STE and CHF. We consider that the 2D-STE technique may provide detailed myocardial functional parameters, and that these novel variables may be useful for detecting CHF in the early stage.

In this study, we hypothesized that 2D-STE-determined myocardial function would be associated with the presence of CHF. We aimed to evaluate the relationship between the existence of CHF and myocardial function assessed by 2D-STE in cats with cardiomyopathy, with and without CHF.

## Materials and Methods

This was a hypothesis-driven, prospective, cross-sectional clinical study. All procedures in this study followed the Guidelines for University Hospital Animal Care of Nippon Veterinary and Life Science University in Japan, and were approved by the ethical committee of our institute (approval number: R2-4).

### Animals

Forty-eight client-owned cats (with cardiomyopathy: *n* = 32; healthy cats: *n* = 16). All cats underwent complete physical examination, electrocardiography, thoracic radiography, blood pressure measurement, and transthoracic echocardiography. Cats were classified into three groups: healthy cats, cardiomyopathy without CHF (CM group), and cardiomyopathy with CHF (CHF group). The healthy cats included cats with no abnormal findings as assessed by the aforementioned examinations. None of the cats were on medication or had a history of clinical signs of heart disease. The CM group included cats clinically diagnosed with HCM or RCM. We diagnosed HCM with echocardiographic evidence of LV hypertrophy and the absence of other diseases known to cause LV hypertrophy. Echocardiographic LV hypertrophy was judged if the LV wall thickness at end-diastole was 6 mm or more, as measured on B-mode echocardiography. LV thickness was measured from the short-axis view, and the mean values of the thickest segment obtained in three consecutive cardiac cycles were used (12). We diagnosed RCM with echocardiographic evidence of left atrial or bi-atrial enlargement and a prominent endomyocardial scar that bridges the interventricular septum and LV free wall. Left atrial enlargement was defined as a left atrial to aortic diameter ratio (LA/Ao) greater than 1.5, obtained from the right parasternal short-axis view, using B-mode echocardiography (13). Right atrial enlargement was judged on the right parasternal long-axis view according to previously published allometric scaling reference intervals (14). Endomyocardial scar findings were macroscopically assessed by B-mode echocardiography. A restricted pattern of LV inflow in cats with RCM was not required because the fusion of E and A waves may prevent detection.

To exclude other forms of feline cardiomyopathy (2), we checked for normal or near-normal LV systolic function, according to previously published allometric scaling reference intervals (15). We excluded cats that had systolic blood pressure >160 mmHg (non-invasive oscillometric method), or systemic or other cardiovascular diseases, including dehydration and myocarditis.

The CHF group included cats that had at least one echocardiographic or radiographic finding providing evidence of left heart failure, such as pulmonary edema, pleural effusion, and the associated clinical signs. Cats with tricuspid regurgitation >2.7 m/s were diagnosed as having pulmonary hypertension (5, 11).

### Echocardiography

Standard 2D and Doppler examinations were performed by a single trained investigator (RS) using a Vivid E95 echocardiography scanner (GE Healthcare) and a 12S transducer (GE Healthcare). Lead II ECG was recorded simultaneously and was displayed on the images. All echocardiographic data were obtained from at least five consecutive cardiac cycles in sinus rhythm in non-sedated cats that were manually restrained in the right and left lateral recumbent positions. Echocardiographic images were analyzed by a single observer (HK) on a separate day from the examination, using an offline workstation (EchoPAC PC, Version 204, GE Healthcare). LA/Ao, end-diastolic interventricular septal thickness, end-diastolic LV free-wall thickness, end-diastolic LV internal diameter (LVIDd), end-systolic LV internal diameter, and fractional shortening (FS) were measured from the right parasternal short-axis view at the level of the chordae tendineae level. Trans-mitral inflow was obtained from the left apical four-chamber view using the pulsed wave Doppler method, and the peak velocity of the early diastolic wave (E-wave) and peak velocity of the late diastolic wave (A-wave) were measured. The E-wave to A-wave ratio (E/A) was also evaluated. In cats whose E and A waves were fused, these values were not used. The end-diastolic right ventricular (RV) internal dimension (RVIDd), end-diastolic RV free wall thickness (RVFWd), and tricuspid annulus plane systolic excursion (TAPSE) were measured using B-mode echocardiography from the left apical four-chamber view modified for right heart measurement (14, 15). The right atrial diameter was measured using the B-mode echocardiography from the mid-point of the interatrial septum to the right atrial lateral wall in the cranial–caudal plane and parallel to the tricuspid valve annulus at end-systole (15). The acceleration-time-to-ejection-time ratio of the pulmonary artery was also calculated from the right parasternal short-axis view of the LV (3). Systolic and early diastolic myocardial velocity of the septal mitral annulus (s' and e', respectively) and the lateral tricuspid annulus (RV s' and RV e', respectively) were obtained by pulsed wave, based on the tissue Doppler method, from the left apical four-chamber view and the left apical four-chamber view modified for right heart measurement (16). Trans-mitral E-wave velocity to early diastolic myocardial velocity of the septal mitral annulus ratio (E/e') was also evaluated. For all analyses, the mean values of three consecutive cardiac cycles from high-quality images were used.

### Two-Dimensional Speckle Tracking Echocardiography

High-quality images for 2D-STE analysis were carefully obtained by the same investigator (RS) using the same echocardiographic system and the same transducer. To evaluate LV myocardial deformations, a right parasternal short-axis view of the left ventricle at the papillary muscles, mitral valve, and apical level of the left ventricle (PM, MV and AP, respectively) and a left apical four-chamber view were obtained in this study. A left apical four-chamber view modified for right heart measurement was also obtained to analyze the right myocardial deformations (11). We measured the peak global strain in the longitudinal and circumferential directions (SL and SC, respectively), which were measured at both ventricles (LV-SL and RV-SL, respectively) (11). For RV deformations, we assessed only the RV lateral wall segments. SC was measured at the papillary muscle, mitral valve, and apical levels of the left ventricle (SC-PM, SC-MV, and SC-AP, respectively) (9, 17). The observer variability of 2D-STE analysis in our laboratory has been described in our previous studies (6, 8, 9, 11). All 2D-STE analyses were performed on a separate day from the examination by a single trained observer (HK) using an offline EchoPAC workstation. The outline of the feline 2D-STE analysis has been described previously (6–9, 12). The mean values of the measurements from three consecutive cardiac cycles from high-quality images were used in all analyses.

### Statistical Analysis

Data are reported as median and interquartile range. Statistical analyses were performed using commercially available software (R 2.8.1; https://www.r-project.org/). The normality of the data distribution was tested using the Shapiro–Wilk test. Continuous variables were compared among groups using one-way analysis of variance or the Kruskal–Wallis test, whichever was appropriate. When a statistically significant difference was detected among the three groups, multiple comparisons were performed using the Steel–Dwass test or Tukey's *post-hoc* test. Logistic regression analysis was used to evaluate the relationship between the existence of CHF and echocardiographic indices (healthy cats + CM vs. CHF group, CM vs. CHF group). Variables with *P* < 0.15 in the univariate models, were included in the multivariate models. Receiver operating characteristic (ROC) curve analysis was performed to assess the diagnostic accuracy of each variable, to detect the presence of CHF. The area under the ROC curve (AUC) was used as a summary measure for diagnostic accuracy and was reported with 95% confidence intervals (CIs). Diagnostic cutoffs for each variable were chosen based on the highest of various combinations of sensitivity and specificity, using Youden's index (18). Multivariable ROC analysis was performed using the variables that were significant (*P* < 0.05) in the univariate ROC analysis to identify the combination of variables that best detected the presence of CHF. The AUC was reported with 95% CIs, and was considered to have high accuracy if it was > 0.9, moderate accuracy if it was 0.7–0.9, and low accuracy if it was 0.5–0.7. For this purpose, different submodels were tested against the full model by taking one submodel at a time as a reference model. The significance level was set at *P* < 0.05.

## Results

### Demographic Data

The demographic data and results of the physical examinations are summarized in Table 1. The CM group included 18 HCM cats and one RCM cat, and there were no cats with pulmonary hypertension. The CHF group included four HCM cats and nine RCM cats, and there were four cats with pulmonary hypertension (*P* < 0.01).

**Table 1:** Clinical characteristics in cats with cardiomyopathy and healthy controls.

**Variables**	**Healthy cats (*n =* 16)**	**CM (*n =* 19)**	**CHF (*n =* 13)**
Age (month)	65 (29–139)	48 (34–76)	97 (46–133)
Sex (male/female)	9/7	12/7	8/5
Body weight (kg)	4.2 (3.6–4.9)	3.9 (3.4–4.2)	4.6 (4.1–5.5)
Heart rate (bpm)	198 (176–225)	170 (158–199)	209 (184–233)
Diagnosis		HCM = 18 RCM = 1	HCM = 4 RCM = 9
CHF (present/past)	0/0	0/0	8/4
Pulmonary hypertension	0 (0%)	0 (0%)	4 (31%)

*CM, cardiomyopathy; CHF, congestive heart failure. Continuous variables were displayed as median (interquartile range)*.

### Echocardiography

Echocardiographic variables are summarized in Table 2. FS was significantly lower in the CM and CHF groups than in the healthy cats (*P* < 0.05). E/e' was significantly higher in the CM and CHF groups than in the healthy cats (*P* < 0.05). LA/Ao, E/A, right atrial diameter, RVIDd, and RVFWd were significantly higher in the CHF group than in the healthy cats (*P* < 0.05). TAPSE and RV s' were significantly decreased in the CHF group as compared to the healthy cats (*P* < 0.05). LA/Ao, LVIDd, and E/A were significantly higher in the CHF group than in the CM group (*P* < 0.05). TAPSE was significantly lower in the CHF group than in the CM group (*P* < 0.05).

**Table 2:** Results of conventional echocardiographic indices in cats with cardiomyopathy and healthy controls.

**Variables**	**Healthy cats (*n =* 16)**	**CM (*n =* 19)**	**CHF (*n =* 13)**
LA/Ao	1.4 (1.2–1.5)	1.5 (1.1–1.5)	2.2 (1.8–3.0) ^*^^†^
LVIDd (mm)	14.2 (13.6–15.6)	13.3 (12.6–14.8)	16.2 (13.7–17.5)^†^
FS (%)	49.8 (41.3–53.0)	41.0 (37.1–43.4)^*^	37.3 (27.9–52.0)^*^
E/A	0.8 (0.8–1.2)	0.9 (0.7–1.2)	3.4 (2.5–3.8)^*^^†^
E/e'	7.9 (7.3–10.1)	14.4 (10.7–19.7)^*^	12.7 (11.7–17.4)^*^
RAD (mm)	9.2 (8.4–10.8)	10.6 (8.1–11.9)	10.8 (10.2–13.4)^*^
RVIDd (mm)	5.7 (4.7–6.4)	4.7 (3.6–6.6)	7.7 (5.6–9.9)^*^^†^
RVFWd (mm)	1.7 (1.6–2.0)	2.0 (1.8–2.2)	2.3 (2.1–2.5)^*^
TAPSE (mm)	9.4 (7.8–11.1)	8.2 (7.6–9.5)	6.6 (5.5–8.2)^*^^†^
RV s' (cm/s)	10.5 (8.6–13.2)	9.4 (8.2–11.0)	7.9 (6.2–9.7)^*^

*CM, cardiomyopathy; CHF, congestive heart failure; LA/Ao, left atrial-to-aortic diameter ratio; LVIDd, end-diastolic LV internal diameter; FS, fractional shortening; E/A, peak velocity of the early diastolic wave-to-peak velocity of the late diastolic wave ratio; E/e', peak velocity of the early diastolic wave-to-early diastolic myocardial velocity of the septal mitral annulus ratio; RAD, right atrial diameter; RVIDd, end-diastolic right ventricular internal dimension; RVFWd, end-diastolic right ventricular free wall thickness; TAPSE, tricuspid annulus plane systolic excursion; RV s', systolic myocardial velocity of the lateral tricuspid annulus. Continuous variables were displayed as median (interquartile range)*.

* *The value is significantly different from the healthy cats (P < 0.05)*.

† *The value is significantly different from that of cardiomyopathy group (P < 0.05)*.

### Two-Dimensional Speckle Tracking Echocardiography

**Figure 1:**
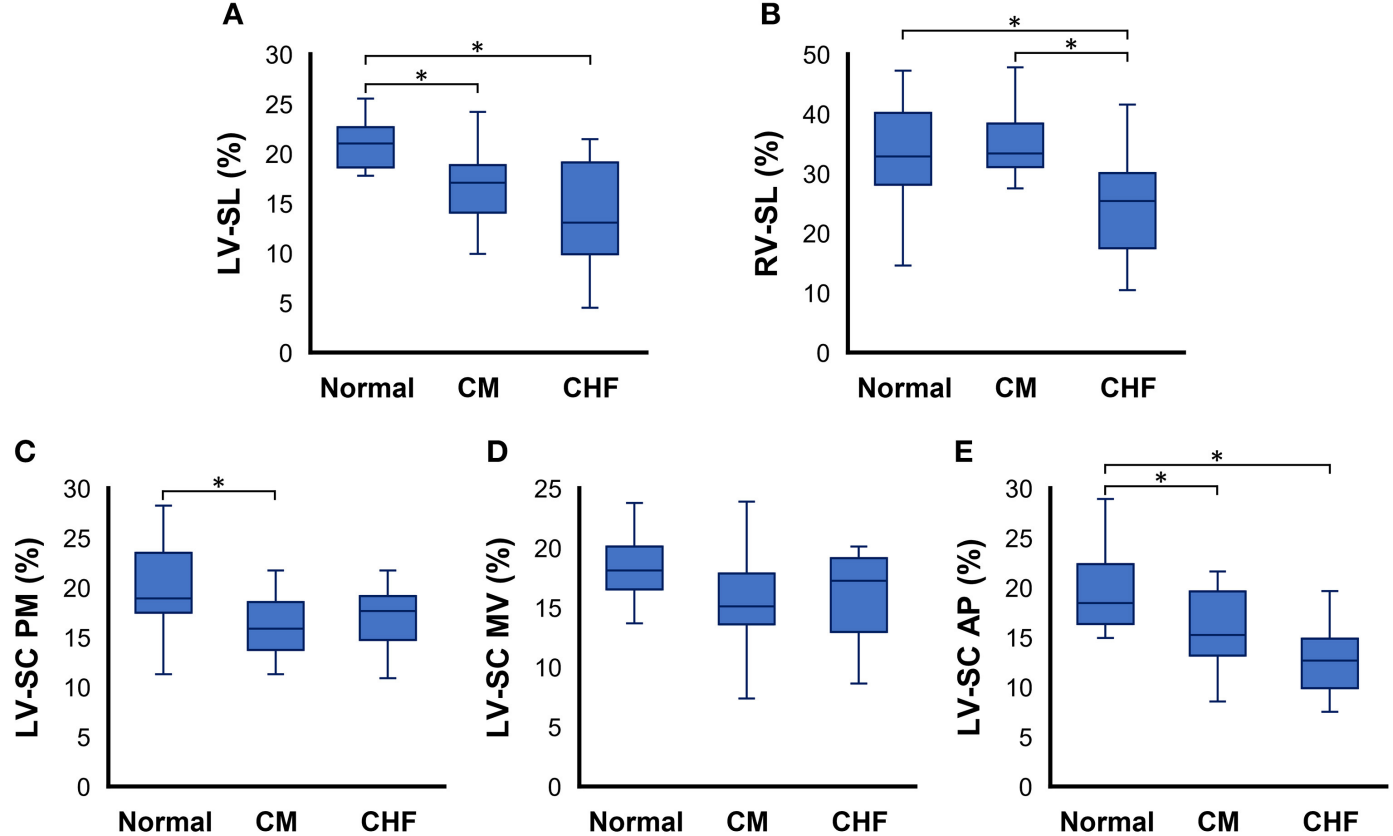
Box and whisker plots of two-dimensional speckle tracking echocardiography indices in cats with cardiomyopathy. **(A)** left ventricular longitudinal strain (LV-SL), **(B)** right ventricular longitudinal strain (RV-SL), **(C)** left ventricular circumferential strain (LV-SC) at the papillary muscles level of the left ventricle (PM), **(D)** LV-SC at the mitral valve level of the left ventricle (MV), and (E) LV-SC at the apical level of the left ventricle AP, respectively. ^*^The value is significantly different between groups (*P* < 0.05).

The results of the 2D-STE variables are summarized in Table 3 and Figure 1. LV-SL (Figure 1A) and LV-SC AP (Figure 1E) were significantly decreased in the CM and CHF groups as compared to the healthy cats (*P* < 0.05). LV-SC PM (Figure 1C) was significantly lower in the CM group than in the healthy cats (*P* < 0.05). RV-SL (Figure 1B) was significantly decreased in the healthy cats and CM groups as compared to that in the CHF group (*P* < 0.05).

**Table 3:** Significant variables in the logistic regression analysis.

**Variables**	**Univariate analysis**		**Multivariate analysis**	
	**Odds ratio (95%CI)**	***P***	**Odds ratio (95%CI)**	***P***
**Healthy cats + CM vs CHF**				
LA/Ao	1.44 (1.16–1.78)	<0.01	1.40 (1.12–1.75)	<0.01
RVIDd	1.06 (1.02–1.10)	<0.01		
LV-SC AP	1.44 (1.13–1.83)	<0.01	1.59 (1.06–2.36)	0.02
RV-SL	2.80 (1.44–5.43)	<0.01		
**CM vs CHF**				
LVIDd	1.06 (1.01–1.12)	0.02		
RVIDd	1.05 (1.01–1.09)	0.01	1.07 (1.00–1.13)	0.01
LV-SC AP	1.30 (1.02–1.65)	0.03		
RV-SL	2.24 (1.16–4.35)	<0.01	1.34 (1.07–1.68)	0.01

*CM, cardiomyopathy; CHF, congestive heart failure; CI, confidence interval; LA/Ao, left atrial-to-aortic diameter ratio; RVIDd, end-diastolic right ventricular internal dimension; LV-SC AP, peak global strain in the circumferential direction at the level of the apical levels of the left ventricle; RV-SL, peak global strain in the longitudinal direction of the right ventricle; LVIDd, end-diastolic LV internal diameter; RVIDd, end-diastolic right ventricular internal dimension. Continuous variables were displayed as median (interquartile range)*.

### Logistic Regression Analysis

The significant variables in the logistic regression analysis are summarized in Table 3. Logistic regression analysis in the healthy cats + CM vs. CHF group showed a significant difference in terms LA/Ao, RVIDd, LV-SC AP, and RV-SL in univariate analysis (*P* < 0.01). LA/Ao and LV-SC AP also showed significant differences in multivariate analysis (*P* < 0.01 and *P* = 0.02, respectively). Logistic regression analysis in the CM vs. CHF group showed a significant difference in LVIDd, RVIDd, LV-SC AP, and RV-SL in univariate analysis (*P* = 0.02, *P* = 0.01, *P* = 0.03, and *P* < 0.01, respectively). RVIDd and RV-SL also showed significant differences in multivariate analysis (*P* = 0.01).

The results obtained by using the ROC curve for variables that were significant in the logistic regression analysis are summarized in Table 4. In the healthy cats + CM vs. CHF group showed high AUC, sensitivity, and specificity in LA/Ao, RVIDd, LV-SC AP, and RV-SL. The ROC curve for the CM vs. CHF group showed a significantly high AUC, sensitivity, and specificity for LVIDd, RVIDd, LV-SC AP, and RV-SL.

**Table 4:** Results of receiver operating characteristic curves of the significant variables in the logistic regression analysis.

**Variables**	**AUC (95%CI)**	**Cutoff**	**Sensitivity**	**Specificity**
**Healthy cats + CM vs. CHF**				
LA/Ao	0.87 (0.71–1.00)	2.07	0.77	1.00
RVIDd	0.78 (0.61–0.96)	6.62	0.77	0.80
LV-SC AP	0.84 (0.72–0.97)	15.16	0.92	0.74
RV-SL	0.86 (0.72–1.00)	24.09	0.77	0.91
**CM vs. CHF**				
LVIDd	0.74 (0.56–0.95)	16.2	0.54	1.00
RVIDd	0.80 (0.63–0.96)	6.85	0.69	0.84
LV-SC AP	0.76 (0.59–0.93)	15.16	0.92	0.58
RV-SL	0.86 (0.71–1.00)	24.09	0.77	0.90

*CM, cardiomyopathy; CHF, congestive heart failure; AUC, area under the curve; CI, confidence interval; LA/Ao, left atrial-to-aortic diameter ratio; RVIDd, end-diastolic right ventricular internal dimension; LV-SC AP, peak global strain in the circumferential direction at the apical level of the left ventricle; RV-SL, peak global strain in the longitudinal direction of the right ventricle; LVIDd, end-diastolic LV internal diameter; RVIDd, end-diastolic right ventricular internal dimension*.

## Discussion

Our study demonstrated that some echocardiographic parameters, including myocardial strain assessed by 2D-STE, are useful for detecting CHF in cats. Specifically, LA/Ao and LV-SC AP derived by 2D-STE may be associated with the progression of CHF in apparently healthy cats. Furthermore, RVIDd and RV-SL derived by 2D-STE might lead to CHF onset in asymptomatic cats with cardiomyopathy. The ROC curves revealed that these parameters had a high AUC with sufficient sensitivity and specificity. We concluded that evaluation of 2D-STE-derived myocardial strain in addition to conventional parameters showed a significant association with the existence of CHF in cats and may be useful tools for the early detection of CHF in these cats.

In this study, left atrial enlargement measured by LA/Ao was significantly higher in the CHF group and performed well in supporting the diagnosis of CHF. Our results agreed with those of previous studies that have reported that LA/Ao is a significant predictor and prognostic indicator of the development of CHF in cats (4, 19, 20). Left atrial enlargement is currently considered a morphophysiological expression of LV diastolic dysfunction, with increasing left atrial size corresponding to progressively worse LV diastolic function and atrial hypertension (21, 22). The higher LA/Ao values observed in the CHF groups in this study reflect diastolic dysfunction that is attributable to cardiomyopathy and increased left atrial pressure, due to congestion pathophysiology. Left atrial enlargement measured by LA/Ao will continue to be a simple and readily available echocardiographic finding that determines the severity of the increase in left atrial pressure and supports the clinical diagnosis of left-sided CHF.

In addition to the left atrium parameter, LV myocardial circumferential strain measured by LV-SC AP was lower in the CHF group than in the CM group. In addition, logistic regression analysis comparing healthy cats + CM vs. CHF revealed that LA enlargement and depressed LV apical myocardial function may be associated with the progression to CHF in apparently healthy cats. Circumferential deformations play an important role in cardiac pump function in humans and dogs with various heart diseases (23–25), and LV myocardial contractions that are impaired in the longitudinal direction are compensated for by circumferential shortening in subclinical patients with cardiovascular risk factors (26). Previous studies on cats have demonstrated that longitudinal strain had already deteriorated in the early stages of cardiomyopathy in cats (11, 12), and circumferential deformations differ according to myocardial compensation. In our results, circumferential deformations differed among LV levels (AP, PM, MV). Apical deformation was lower in cats with cardiomyopathy. Apical deformations have been reported as a major factor in torsion and relaxation of the entire left ventricle (9) thus, early myocardial dysfunction may occur at the apical level of circumferential deformations. The depressed LV-SC AP variables observed in this study may reflect myocardial dysfunction in decompensated patients and may lead to CHF development in these cats. In addition to LA enlargement, decreased LV-SC AP may be a useful tool for detecting CHF in cats.

In the present study, significant findings were also observed in RV echocardiographic indices. Furthermore, a comparison between the CM and CHF groups revealed that RV enlargement and dysfunction might lead to CHF onset in asymptomatic cats with cardiomyopathy. In humans, RV assessment has been shown to have a clinically relevant impact on predicting clinical status and outcome in a variety of cardiovascular diseases (27–31). In veterinary medicine, RV dysfunction has been shown to be a prognostic factor in dogs with arrhythmogenic RV cardiomyopathy and myxomatous mitral valve disease (32, 33). In the present study, RV-SL measured by 2D-STE, which may allow for a more detailed evaluation of myocardial function, showed no significant change in the CM group, but was decreased in the CHF group as compared with the healthy cats. Previous reports have shown no significant differences in RV-SL between asymptomatic HCM cats and healthy cats (10), which was in agreement with our study. The results of cats with CHF suggest that more extensive cardiomyopathy-associated lesions may develop in the right as well as in the left ventricle, and/or that pulmonary hypertension due to pulmonary venous congestion might affect RV function, particularly in cats with advanced stages of heart failure (i.e., CHF condition). A previous case report on RCM demonstrated that RV-SL decreased with the progression of the clinical course of RCM, although it did not decrease in the early stage of this disease (34). Additionally, it has also been reported that, in human HCM patients, a decrease in RV-SL is associated with the development of CHF (35). Therefore, our results suggest that RV dysfunction might occur in cats with CHF and that decreased RV-SL may be a predictor of CHF development in cats with cardiomyopathy.

In this study, RV dilatation, as assessed by RVIDd, showed a significant association with the presence of CHF. We previously described the RV adaptation mechanism during the progression of pulmonary hypertension, which would induce RV dilatation to maintain cardiac output (36). Only cats with CHF showed significant RV dysfunction and dilatation in our study population, suggesting that the RV adaptation mechanism might be decompensated in these cats. Therefore, our results suggest that RV dilatation, as assessed by RVIDd, as well as decreased RV-SL might provide additional information for CHF development in cats with cardiomyopathy.

This study had several limitations. First, because it was a non-invasive clinical investigation, we had no access to histopathological findings to make a definitive diagnosis and assess myocardial histopathological alterations. Second, we could not consider the influence of medication on the values assessed by 2D-STE. Because cats with CHF showed signs of heart failure, some cats had received drugs that could affect myocardial performance prior to examination. However, medication-controlled cats in the CHF group had the worst myocardial function in this study. Third, the small number of cats in our study may have influenced the statistical power and limited extrapolation of our findings to larger populations. Fourth, recent study has reported that the ratio of pulmonary veins to pulmonary artery might be useful for the detection of cats with CHF (37). Unfortunately, we could not have evaluated the variable due to the lack of appropriate echocardiographic data to measure the variables in this study. The relationship between the variables and the presence of CHF should be compared in the future. Finally, this was a cross-sectional study and included cases with different types of cardiomyopathy and pulmonary hypertension. These confounding factors may influence interpretation of our results.

In conclusion, logistic regression analysis comparing healthy cats + CM vs. CHF revealed that left atrial enlargement and depressed LV apical myocardial function may be associated with the progression from subclinical patients to CHF. Furthermore, a comparison between the CM and CHF groups revealed that RV enlargement and dysfunction might lead to CHF onset in asymptomatic cats with cardiomyopathy. Additionally, the receiver operating characteristic curves revealed that LA/Ao, RVIDd, and 2D-STE-derived apical LV-SC and RV-SL had a high AUC in ROC curve analysis, with sufficient sensitivity and specificity for indicating the presence of CHF. Thus, 2D-STE-derived myocardial strain showed a significant association with the existence of CHF in cats and may therefore be a useful tool for the early detection of CHF in cats with cardiomyopathy. Nevertheless, further studies with larger sample sizes are required to verify our findings.

## Data Availability Statement

The raw data supporting the conclusions of this article will be made available by the authors, without undue reservation.

## Ethics Statement

The animal study was reviewed and approved by Ethical Committee for Laboratory Animal Use of the Nippon Veterinary and Life Science University. Written informed consent was obtained from the owners for the participation of their animals in this study.

## Author Contributions

RS and TS performed the concept/design, data acquisition, interpretation, critical revision of the article, and drafting the article. YY and HKa performed the data acquisition and data analysis, and summarized the clinical data. TT, HM, and HKo performed data acquisition and interpretation, and provided the academic direction. All authors have read the final version of this paper and approved submission.

## Funding

This work was partially supported by the Japan Society for the Promotion of Science (JSPS), Grant Number 20K15667.

## Conflict of Interest

The authors declare that the research was conducted in the absence of any commercial or financial relationships that could be construed as a potential conflict of interest.

## Publisher's Note

All claims expressed in this article are solely those of the authors and do not necessarily represent those of their affiliated organizations, or those of the publisher, the editors and the reviewers. Any product that may be evaluated in this article, or claim that may be made by its manufacturer, is not guaranteed or endorsed by the publisher.

## Abbreviations

2D-STE: two-dimensional speckle tracking echocardiography
AP: apical level of the left ventricle
AUC: area under the receiver operating characteristic curve
A-wave: peak velocity of the late diastolic wave
CHF: congestive heart failure
Cis: confidence intervals
E/A: E-wave to A-wave ratio
E/e': trans-mitral E-wave velocity to early diastolic myocardial velocity of the septal mitral annulus ratio
e': early diastolic myocardial velocity of the septal mitral annulus
E-wave: peak velocity of the early diastolic wave
FS: fractional shortening
HCM: hypertrophic cardiomyopathy
LA/Ao: left atrium to aortic diameter ratio
LV: left ventricular
LVIDd: end-diastolic LV internal diameter
MV: mitral valve level of the left ventricle
PM: papillary muscle level of the left ventricle
RCM: restrictive cardiomyopathy
ROC: receiver operating characteristic
RV: right ventricular
RV e': early-diastolic myocardial velocity of the lateral tricuspid annulus
RV s': systolic myocardial velocity of the lateral tricuspid annulus
RVFWd: end-diastolic RV free wall thickness
RVIDd: end-diastolic right ventricular internal dimension
s': systolic myocardial velocity of the septal mitral annulus
SC: circumferential strain
SL: longitudinal strain
TAPSE: tricuspid annulus plane systolic excursion.
